# Spring-mediated cranioplasty for sagittal craniosynostosis

**DOI:** 10.3171/2021.1.FOCVID2060

**Published:** 2021-04-01

**Authors:** Christopher L. Kalmar, Jordan W. Swanson, Sameer Shakir, Alexander M. Tucker, Benjamin C. Kennedy, Phillip B. Storm, Gregory G. Heuer, Scott P. Bartlett, Jesse A. Taylor, Shih-Shan Lang

**Affiliations:** Divisions of 1 Plastic and Reconstructive Surgery and; 2 Neurosurgery, Children's Hospital of Philadelphia, Pennsylvania

**Keywords:** sagittal, craniosynostosis, springs, technique, video

## Abstract

Spring-mediated cranioplasty is a useful treatment modality for correcting scaphocephalic head shape in sagittal craniosynostosis because it is less invasive than whole-vault cranioplasty and offers durable morphologic outcomes. Herein, the authors provide a multimedia demonstration of alternative operative approaches for spring-mediated cranioplasty for sagittal craniosynostosis.

The video can be found here: https://vimeo.com/511256259

## Transcript

**0:25 Goals**. The primary goals of spring-mediated cranioplasty for the correction of sagittal craniosynostosis is to sufficiently transversely expand the parietal bones to reverse midvault craniocerebral disproportion, and thus to correct head shape deformity and prevent associated neurocognitive impairment.

**0:46 Indications**. Spring-mediated cranioplasty is typically our institution's treatment of choice for infants presenting with isolated, nonsyndromic sagittal craniosynostosis prior to age 5 months depending on phenotypic severity and gestational age.

**1:03 Preoperative Planning**. Newborns with suspected sagittal craniosynostosis typically undergo low-dose noncontrast head CT imaging to confirm the diagnosis and evaluate for any other suture closures with a joint consultation with neurosurgery and plastic surgery preoperatively, as well as with ophthalmology and genetics. Most infants undergo spring-mediated cranioplasty at 3–4 months of age, and our technique has been previously reported.^1^

**1:25 Ex Vivo Cranial Spring Preparation**. Stainless steel wire of 0.051-inch diameter is manually bent in the shape of a U, with two 90° bends to create 1-cm footplates opposite the U bend. The cranial springs are loaded onto a force dynamometer at a 1.5-cm width in order to simulate force exertion across the planned strip craniectomy defect. Cranial springs are created at varying lengths (typically 8–16 cm), varying bend widths, and with an alternative wire diameter (0.059 inch) in order to alter the resultant spring force. The various cranial springs are subsequently packaged and sterilized for intraoperative use.

**1:57 Overview**. Spring-mediated cranioplasty can be performed either supine or prone, depending on surgeon preference. The cranium is exposed by one of two incision types: 1) two transverse incisions equidistant between the anterior and posterior fontanelles, or 2) midparietal scalp chevron incision that could be incorporated into a bicoronal incision in the future, if further open-vault expansion is needed. Subgaleal drains are not typically used.

**2:28 Prone Positioning**. Advantages of placing the patient prone include easy access to the mid- and posterior vault. We have abandoned the “sphinx” position at our institution in order to avoid potential risks associated with cervical spine stability, impaired venous return, and spinal cord ischemia.

**2:44 Linear Incisions**. Two linear 3- to 4-cm transverse incisions are made posterior to the anterior fontanelle and anterior to the posterior fontanelle after subcutaneous infiltration with bupivacaine with epinephrine. The advantage of placing two linear incisions is easier access to the most distal anterior and posterior aspects of the sagittal suture for performing the craniectomy and accurately placing the distractor springs. The disadvantage of placing two linear incisions is potential wound healing issues: if the patient undergoes any additional bicoronal incision in the future, there will be three parallel incisions.

**3:24 Scalp Elevated**. A subgaleal plane is made with Metzenbaum scissors between the fontanelles.

**3:36 Burr Hole and Strip Craniectomy**. If performing spring-mediated cranioplasty through two separate transverse incisions, a burr hole is made in the midline at the most inferior incision first, in order to prevent pooling of blood. This may be the anterior aspect or posterior aspect of the skull, depending on patient positioning. The second burr hole is made in the midline at the superior incision, which also may be the anterior aspect or posterior aspect of the skull, depending on patient positioning.

In this patient example, the patient has been positioned prone; therefore, the first inferior (here anterior) burr hole is made close to the anterior fontanelle. The Sonopet ultrasonic aspirator is used to perform a craniectomy from the anterior burr hole to the anterior fontanelle. The second burr hole is placed at the most superior (here posterior) incision after the anterior strip craniectomy is completed, and the Sonopet is used to perform a craniectomy between the anterior and posterior burr holes. And finally, the Sonopet is used to perform a craniectomy from the superior/posterior burr hole to the posterior fontanelle and lambdoid sutures. In total, three thin craniectomy strips with a width of usually 1.5 cm or less are removed. Hemostasis is ensured with use of gelatin human thrombin matrix as needed.

In this patient example, the patient has been positioned supine. If performing spring-mediated cranioplasty through one chevron incision, a burr hole is placed equidistant between the two fontanelles, and only two strip craniectomy pieces are removed.

**5:09 Spring Selection**. Two or three cranial springs are selected intraoperatively by the craniofacial surgeon for each case. As previously described, spring selection is based on spring variables including length, wire thickness, and U bend—factors that alter the resultant transverse distraction force across the suturectomy site. Most commonly, three springs are utilized at the anterior, midvault, and posterior positions.

A third spring is avoided if insertion leads to 1) excessive crowding due to spring overlap, 2) excessive prominence in the sagittal plane, 3) spring dislodgment, and/or 4) undue soft-tissue tension.

**5:52 Out-of-Plane Bending**. Out-of-plane spring bending is performed with a Tessier bone bender, and the footplates are modified to fit along the craniectomy site. Out-of-plane bending is important to align the spring contour with the cranial bone on which it rests, mitigating spring prominence that could lead to spring dislodgment, delayed healing of the scalp, and/or seroma.

**6:16 Spring Placement**. Matched osteotomy notches are made using a Lempert rongeur along each margin of the strip craniectomy; then each spring is inserted with footplates engaged into the notches. Springs are placed 1 cm posterior to the coronal sutures, 1 cm anterior to the lambdoid sutures, and if a third spring is used, it is placed at the parietal bone midpoint.

**6:57 Morselized Cranial Bone Replaced**. The wound is then copiously irrigated, and hemostasis is ensured. The previously removed sagittal suture is morselized and placed back into the osteotomy site and secured with gelatin human thrombin matrix in order to allow for a scaffold to facilitate bone growth deep to the springs.

**7:17 Closing**. The incisions are closed with 3-0 braided absorbable suture in the galeal layer, and then with 4-0 transcutaneous plain gut suture.

### 7:27 Alternative Considerations

#### Supine Positioning

The procedure can also be performed efficiently with the patient placed supine. Advantages of this position include easier access to the frontal region for more frontal pathology, easier access to the airway for anesthesia, and theoretical decreased risk of pressure-induced injury to the face, especially the orbits.

#### Chevron Incision

The procedure can also be performed with a single chevron incision. Advantages of this incision include the ability to later extend the incision to a full cranial incision, if needed in the future for whole-vault cranial remodeling.

Since Claus Lauritzen's initial clinical description in 1998, cranial spring technology has become an increasingly useful treatment modality for more minimally invasive approaches to the craniofacial skeleton when compared to conventional open techniques—especially for the correction of sagittal craniosynostosis-induced scaphocephaly in young infants—and offers durable morphological outcomes.^2,3^ Various craniofacial units across Europe and the United States have published not only comparable long-term efficacy in intracranial pressure risk reduction and scaphocephalic head shape correction, but also improved perioperative outcomes when compared to conventional open cranial vault remodeling techniques.^4–8^ As mounting evidence suggests improved neurodevelopmental outcomes of interventions performed at an earlier age, spring-mediated cranioplasty may offer an ideal balance between minimizing perioperative risks while optimizing head shape.^9^ Head shape changes affected by spring-mediated cranioplasty are both direct (biparietal widening) and indirect (occipital bulleting and frontal bossing usually resolve with growth after midvault expansion is performed). The trade-offs of spring-mediated cranioplasty largely relate to a short second surgery for spring removal, although this can usually be accomplished as an outpatient surgical procedure. Blood transfusion is needed in a small portion of cases, particularly in patients with lower preoperative hemoglobin. Future directions will prospectively compare the perioperative and postoperative outcomes of spring-mediated cranioplasty to existing techniques, including open cranial vault remodeling and endoscopic strip craniectomy with postoperative helmeting, in order to definitively determine the comparative effectiveness of these modalities in sagittal craniosynostosis.

### 8:10 Technical Pearls

- Spring-mediated cranioplasty performed at a younger age leads to increased change in cephalic index by maximizing the compliance of a viscoelastic skull. Expanding parietal bones at a younger age tends to lead to resolution of frontal bossing and occipital bulleting.
- Three springs enables a larger total force to be applied than two springs, and distributes the force more gradually along bone contact points, which appears to drive greater outcome consistency.
- The degree of cranial expansion observed 1 month postoperatively serves as a gross clinical endpoint of expansion; however, ongoing remodeling of frontal bossing or occipital bulleting can be clinically observed for several months thereafter.
- In our experience, greater distracting forces may be achieved when utilizing wider, thicker springs independent of spring length and degree of compression, which may be appropriate for older infants.

## Acknowledgments

We would like to acknowledge Dr. Laura S. Humphries for her expertise and demonstration custom bending cranial springs.

## Author Contributions

Primary surgeon: Lang, Swanson, Storm, Bartlett, Taylor. Assistant surgeon: Lang, Taylor. Editing and drafting the video and abstract: Lang, Kalmar, Swanson, Shakir. Critically revising the work: Lang, Kalmar, Swanson, Shakir, Kennedy, Storm, Heuer. Reviewed submitted version of the work: Lang, Kalmar, Swanson, Shakir, Tucker, Kennedy, Storm, Heuer, Taylor. Approved the final version of the work on behalf of all authors: Lang. Supervision: Lang, Heuer, Bartlett.
